# State Sanitarium for Incipient Tuberculosis

**Published:** 1906-11

**Authors:** 


State Sanitarium for Incipient Tuberculosis.—On August 15th last the cornerstone of the new sanitarium for the treatment of incipient tuberculosis was laid. More than 500 physicians from all parts of the State were present. Governor Folk delivered a characteristic address and said that one of the pleasantest duties he had to perform was to sign the bill providing for this institution. Dr. William Porter, of St. Louis, also addressed the assembly. He quoted figures showing the annual economic loss to the State at the present death rate from tuberculosis and the saving which will be effected when the sanitarium is in full operation. He estimated that at present there are 20,000 cases of tuberculosis in Missouri, entailing a loss of $24,000,000 annually. Forty per cent of these cases can be saved. This means a saving of $9,000,000 annually to the State. But untold good will be done by restoring to health some who were marked for the grave and by educating the people to protect themselves from infection. Missouri must not permit this institution to fail in its mission, and the Legislature should appropriate a liberal sum of money for maintaining the work now about to begin.—*Missouri State Medical Journal.*

